# What comprises the plate-like structure between the pancreatic head and the celiac trunk and superior mesenteric artery? A proposal for the term “P–A ligament” based on anatomical findings

**DOI:** 10.1007/s12565-020-00597-1

**Published:** 2021-01-08

**Authors:** Satoru Muro, Wachirawit Sirirat, Daisuke Ban, Yuichi Nagakawa, Keiichi Akita

**Affiliations:** 1 Department of Clinical Anatomy, Tokyo Medical and Dental University, 1-5-45 Yushima, Bunkyo-ku, Tokyo, 113-8510 Japan; 2 Department of Hepatobiliary and Pancreatic Surgery, National Cancer Center Hospital, 5-1-1 Tsukiji, Chuo-ku, Tokyo, 104-0045 Japan; 3 Department of Gastrointestinal and Pediatric Surgery, Tokyo Medical University, 6-1-1 Shinjuku, Shinjuku-ku, Tokyo, 160-8402 Japan

**Keywords:** Mesopancreas, Pancreas, Pancreatic head plexus, P–A ligament, Surgical anatomy

## Abstract

A plate-like structure is located posterior to the portal vein system, between the pancreatic head and roots and/or branches of two major arteries of the aorta: the celiac trunk and superior mesenteric artery. We aimed to clarify the distribution and components of this plate-like structure. Macroscopic examination of the upper abdomen and histological examination of the plate-like structure were performed on 26 cadavers. The plate-like structure is connected to major arteries (aorta, celiac trunk, superior mesenteric artery) and the pancreatic head; it contains abundant fibrous bundles comprising nerves, vessels, collagen fibers, and adipose tissue. Furthermore, it consists of three partly overlapping fibrous components: rich fibrous bundles (superior mesenteric artery plexus) fused to the uncinate process of the pancreas; fibrous bundles arising from the right celiac ganglion and celiac trunk that spread radially to the dorsal side of the pancreatic head and superior mesenteric artery plexus; and fibrous bundles, accompanied by the inferior pancreaticoduodenal artery, entering the pancreatic head. The plate-like structure is the pancreas–major arteries (aorta, celiac trunk, superior mesenteric artery) ligament (P–A ligament). The term “P–A ligament” may be clinically useful and can facilitate comprehensive understanding of the anatomy surrounding the pancreatic head and provide an anatomical basis for further pancreatic surgery studies.

## Introduction

Cancer of the pancreatic head often invades the superior mesenteric artery (SMA) (Nagakawa et al. 1992; Nakao et al. 1996, 2004; Makino et al. 2008; Deshmukh et al. 2010). Between the pancreatic head and the SMA is a plate-like structure that surgeons grasp during pancreaticoduodenectomy to separate the pancreatic head from the SMA or celiac trunk (CeT) to determine the line of incision while preserving important blood vessels.

This plate-like structure, located between the pancreatic head and the CeT/SMA, has been attributed two descriptions (Yoshioka and Wakabayashi 1985; Yi et al. 2003; Gockel et al. 2007; Agrawal et al. 2010; Inoue et al. 2015; Kanhere et al. 2015). First, it was described as the mesopancreas. This description recognizes the plate-like structure as the mesentery of the pancreas (Gockel et al. 2007). However, the term “mesopancreas” describes only the structure between the pancreatic head and the SMA and is problematic because its defined structural boundaries are ambiguous. Furthermore, the term “mesopancreas” is reminiscent of an oncological benefit, although none has been revealed. Second, it has been described as the pancreatic head plexus (Yoshioka and Wakabayashi 1985; Yi et al. 2003). This description focuses on the nerve pathways and is limited to the anatomy of the nervous system. However, this anatomy of the nerve only partially describes the plate-like structure, including its thick fibrous tissue (Yoshioka and Wakabayashi 1985; Yi et al. 2003). Therefore, the terms “mesopancreas” and “pancreas head plexus” have some important implications but do not comprehensively explain the anatomy of the plate-like structure. A more precise anatomical description of this area is therefore sought by those in the fields of surgery, radiology, and pathology.

We propose calling this plate-like structure the “pancreas–major arteries ligament” (P–A ligament) because it lies between the pancreas and major arteries (aorta, CeT, and SMA). The present study aimed to clarify the distribution and components of this plate-like structure and advocate clinically useful terminology. We believe that this term will contribute to further discussions of the anatomy and pancreatic cancer treatments.

## Materials and methods

The study has been approved by the Board of Ethics of Tokyo Medical and Dental University (approval number: M2019-070) and has been performed in accordance with the ethical standards laid down in the 1964 Declaration of Helsinki and its later amendments. The work was performed in agreement with the Japanese Act on Body Donation for Medical and Dental Education 1983.

The cadavers used in the present study were donated to the Department of Anatomy at Tokyo Medical and Dental University in Japan. All of the donors voluntarily declared before their deaths that their remains would be donated as materials for education and study. This voluntary donor system of cadavers is applied throughout Japan, and our study was in complete compliance with the current laws of Japan. All cadavers were fixed by arterial perfusion with 8% formalin and preserved in 30% alcohol.

### Macroscopic anatomy

Twenty-two cadavers (8 men and 14 women; age range at death, 65–97 years; mean age at death, 84.6 years) were used for macroscopic examinations of the whole upper abdomen. Each upper abdomen, including the pancreas, stomach, duodenum, jejunum, liver, spleen, aorta, inferior vena cava, and adjacent tissues, was obtained en bloc from the cadavers. Seven upper abdomens were dissected from the ventral side. The common hepatic artery was cut and removed along with its branches to identify the portal vein (PV). The pancreas was cut on the right borderline of the PV and superior mesenteric vein (SMV), and the body and tail of the pancreas were removed to examine the PV, SMV, and splenic vein. Subsequently, the PV system was removed to observe the plate-like structure posterior to it. The loose connective tissue was precisely removed from the plate-like structure for observation. Two other upper abdomens were dissected from the dorsal side. The CeT and SMA were cut at the root, and the aorta was removed. After removal of the inferior vena cava, the plate-like structure between the pancreatic head and the CeT/SMA was observed along with the inverted right celiac ganglion. The loose connective tissue was precisely removed to observe the components of the plate-like structure. The other 13 upper abdomens were dissected from both the ventral and dorsal sides using the aforementioned procedure.

Two cadavers (one male and one female; age at death, 92 and 96 years, respectively) were used for macroscopic examinations of specimens isolated en bloc. The limited region of the head of the pancreas was obtained en bloc with the surrounding structures, including the duodenum, CeT, SMA, PV, and SMV. Specimens were dissected from both the ventral and dorsal sides. The PV and SMV were removed to show the plate-like structure between the pancreatic head and the CeT/SMA. Subsequently, the loose connective tissue was precisely removed so that the components of the plate-like structure could be observed. Thereafter, non-arterial tissues were removed to reveal arterioles.

### Histology

The fibrous bundles of the plate-like structure located between the pancreatic head and the CeT/SMA were obtained from the cadaveric specimen after dissection to identify the type of tissue included in these fibrous bundles. The fibrous bundles were fixed in 10% formalin, dehydrated, and embedded in paraffin. They were sectioned into 5-μm-thick specimens and stained with hematoxylin and eosin.

Additional two cadavers were used for histological examinations of the transverse section. Their upper abdomens were obtained en bloc, frozen at − 80 °C, and cut into 8-mm-thick transverse sections from which small blocks were obtained for histological analysis. The blocks were fixed in 10% formalin, dehydrated, and embedded in paraffin. Then, 5-μm-thick histological sections were created and stained with hematoxylin and eosin.

## Results

**Fig. 1:**
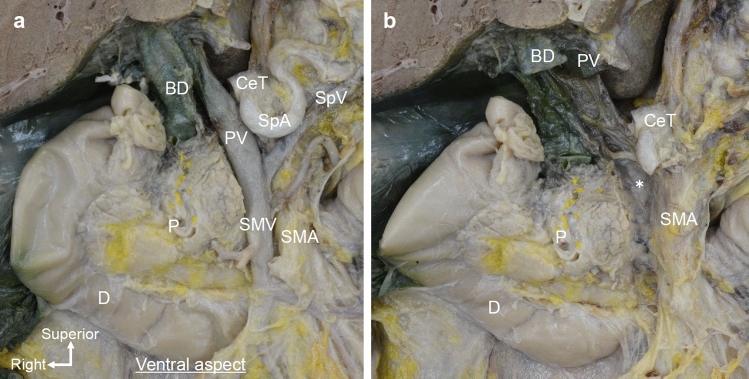
Macroscopic anatomy of the plate-like structure as viewed from the ventral side. **a** Ventral side of the pancreatic head after removal of the body and tail of the pancreas. The PV system, including the PV, SMV, and SpV, was observed. **b** After removal of the PV system, a plate-like structure (*) was observed. It was continuously connected to the pancreatic head and CeT/SMA. *BD* bile duct, *CeT* celiac trunk, *D* duodenum, *P* pancreas, *PV* portal vein, *SMA* superior mesenteric artery, *SMV* superior mesenteric vein, *SpA* splenic artery, *SpV* splenic vein

We examined the anatomy of the pancreatic head and surrounding structures from the viewpoint of pancreaticoduodenectomy (Fig. 1a). The pancreatic head was pulled slightly to the right to simulate the situation during surgery. On the dorsal side of the PV and SMV, a plate-like structure was found to be continuously fused to the uncinate process of the pancreas and CeT/SMA (Fig. 1b). It was contained within a thin fascia that continued from the SMA plexus to the pancreatic fascia.

**Fig. 2:**
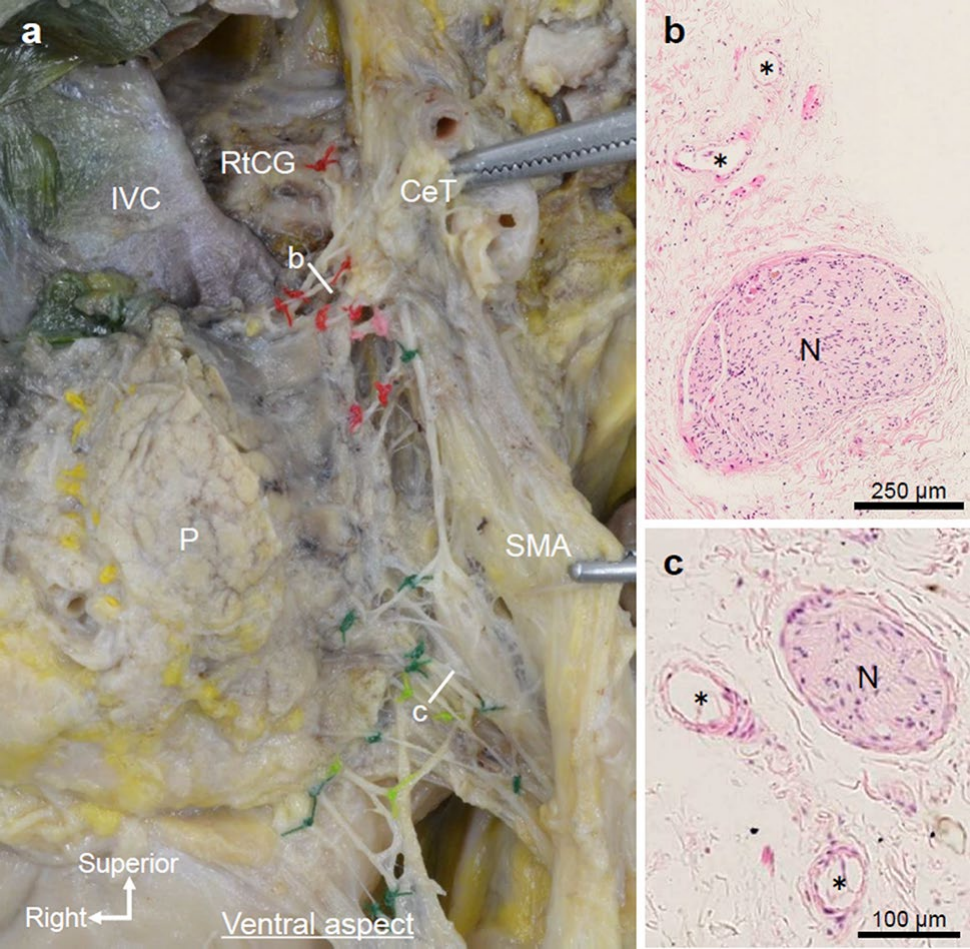
Components of the plate-like structure: abundant fibrous bundles. **a** Ventral side of the same specimen shown in Fig. 1 after dissection of the plate-like structure (marked by * in Fig. 1b). The pancreatic head is pulled slightly to the right to simulate the situation during surgery. Abundant fibrous bundles connecting the RtCG, CeT, SMA, and the uncinate process of the pancreas were observed. **b**, **c** Hematoxylin and eosin staining of the fibrous bundles shown in (a). Nerves, vessels (*), and collagen fibers were observed. *CeT* celiac trunk, *IVC* inferior vena cava, *N* nerve, *P* pancreas, *RtCG* right celiac ganglion, *SMA* superior mesenteric artery

The plate-like structure included abundant fibrous tissue composed of nerves, vessels, collagen fibers, and adipose tissue. After removal of the loose connective tissue from the plate-like structure, abundant fibrous bundles were revealed (Fig. 2a). Most of the fibrous bundles were nerves that arose from the left and right ganglia and ran along the CeT or SMA. Small arteries, and veins were also macroscopically observed in the fibrous bundles. Between the uncinate process and SMA, the fibrous bundles mainly ran along the arteries and formed a network while communicating with each other (SMA plexus). The uncinate process was attached to the SMA plexus, and some fibers were connected between the uncinate process and SMA plexus. When the pancreatic head was pulled to the right, as it is during pancreaticoduodenectomy, the entire fibrous bundle was observed as a tent-shaped structure that pulled on the cranial and caudal parts of the uncinate process. In other words, fibrous bundles run obliquely between the upper part of the uncinate process and CeT or proximal SMA, and between the lower part of the uncinate process and the dorsal side of the distal SMA, which were tent-shaped. The cranial and caudal parts of the uncinate process were attached to relatively thick fibers of the SMA plexus; however, only thin branches entered the pancreatic head between the cranial and caudal parts. Abundant fibrous bundles ran along the inferior pancreaticoduodenal artery (IPDA). Histologically, the fibrous bundles included nerves, vessels, and collagen fibers (Fig. 2b, c).

**Fig. 3:**
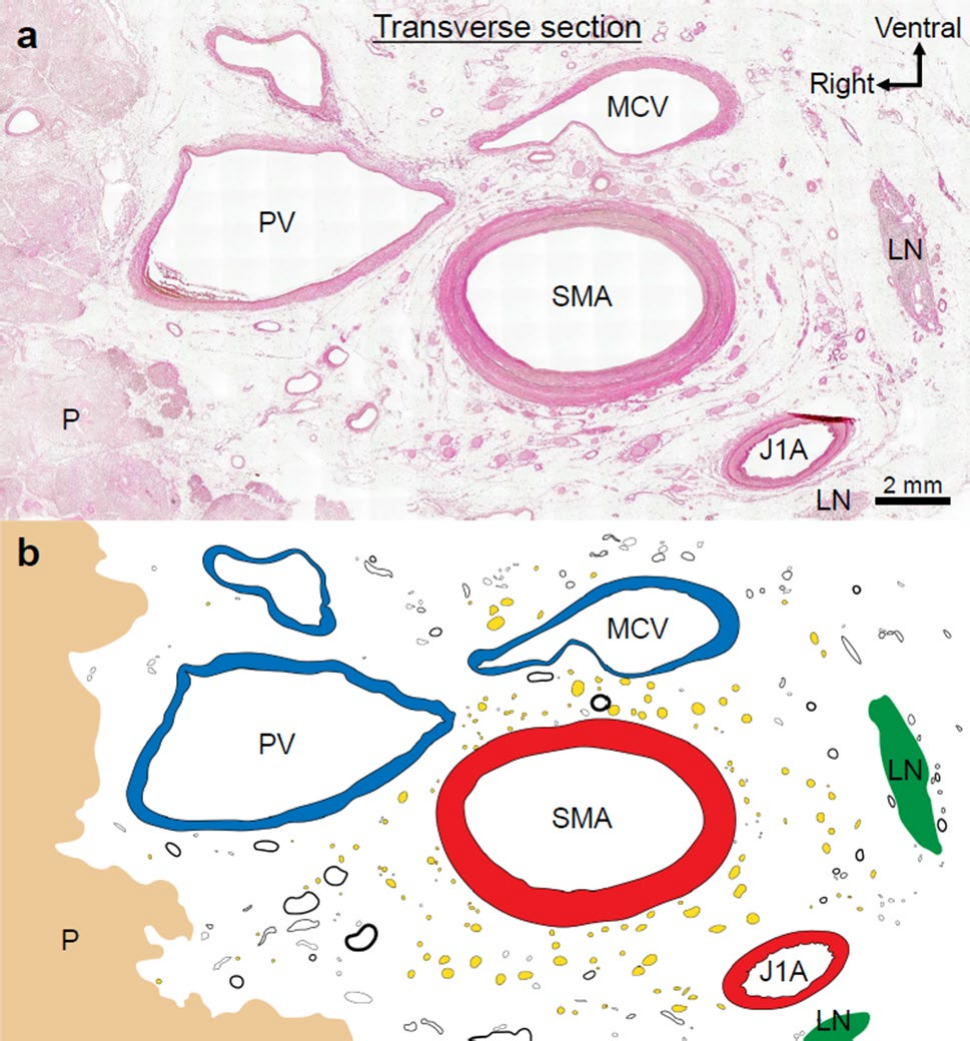
**a** Transverse histological section of the plate-like structure (hematoxylin and eosin stain. **b** Nerves, vessels (arteries, veins, and lymph ducts), collagen fibers, and adipose tissue existed in the region between the uncinate process of the pancreas and the SMA (nerves are indicated by yellow dots; vessels are indicated by circles). Collagen fibers encircled the SMA concentrically and wrapped around the nerves and small vessels in multiple layers to form the SMA plexus. *LN* lymph node, *P* pancreas, *PV* portal vein, *MCV* middle colic vein, *SMA* superior mesenteric artery, *J1A* first jejunal artery

The transverse histological section exhibited nerves, vessels (arteries, veins, and lymph ducts), collagen fibers, and adipose tissue between the uncinate process of the pancreas and SMA (Fig. 3). Large and small vessels were mixed, and the presence of arterioles was confirmed. Thin nerves were observed near the pancreas and thick nerves were observed near the SMA. The SMA was concentrically encircled by the collagen fibers that wrapped around the nerves and small vessels in multiple layers to form the SMA plexus.

**Fig. 4:**
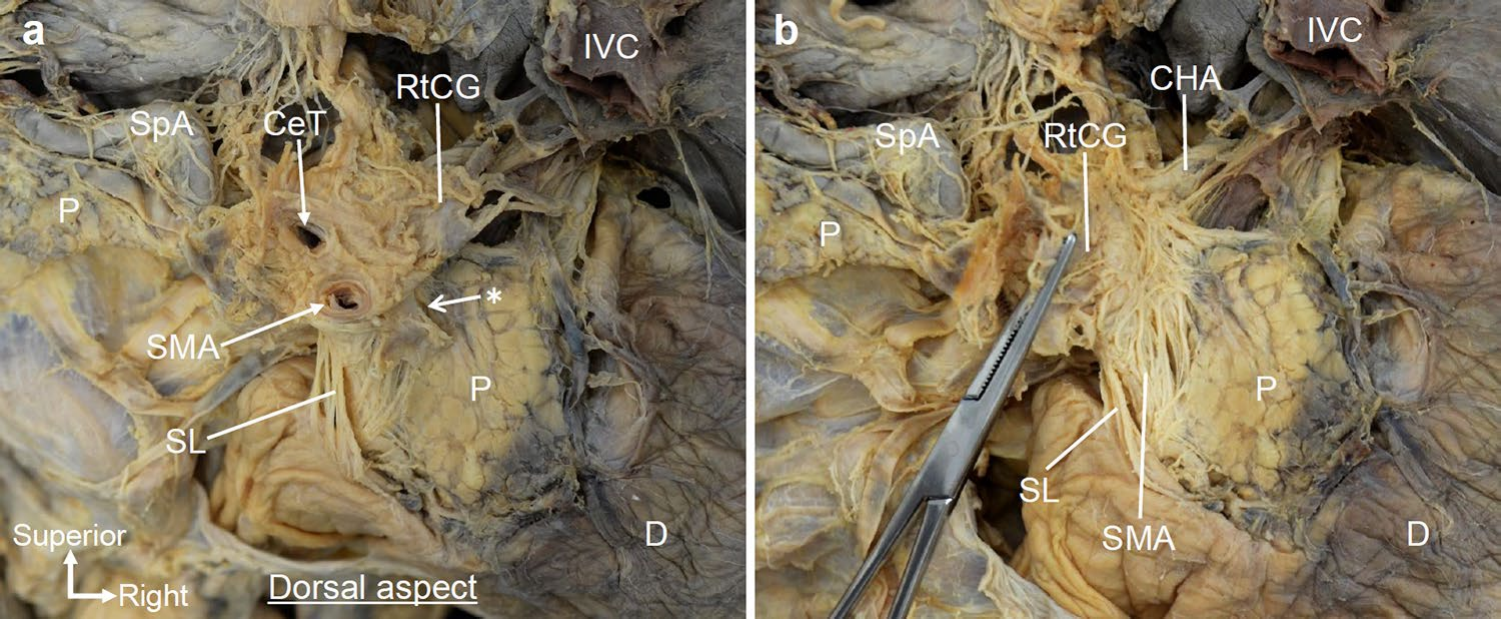
Dorsal view of the macroscopic examination of the fibrous bundles spreading radially to the pancreatic head. **a** Dorsal view of the pancreas after removal of the aorta and IVC. The plate-like structure was located between the pancreatic head and the roots of the CeT and SMA (*). **b** The RtCG was inverted to the left side, and loose connective tissue was removed from the plate-like structure. Fibrous bundles of the plate-like structure were observed between the pancreatic head and the CeT/SMA. These fibrous bundles ran radially from the RtCG and CeT toward the dorsal side of the pancreatic head. *CeT* celiac trunk, *CHA* common hepatic artery, *D* duodenum, *IVC* inferior vena cava, *P* pancreas, *RtCG* right celiac ganglion, *SL* suspensory ligament of duodenum (Treitz’ ligament), *SMA* superior mesenteric artery, *SpA* splenic artery

Macroscopic examination of the dorsal side showed fibrous bundles of the plate-like structure. The right celiac ganglion was located to the right of the CeT, and fibers protruded near the roots of the CeT and SMA (Fig. 4a). The roots of the CeT and SMA were surrounded by numerous fibers including the nerves and collagen fibers. Some of the fibrous bundles originating from the right celiac ganglion and the roots of the CeT and SMA were distributed radially to the dorsal side of the pancreatic head, and others descended toward the SMA (Fig. 4b).

**Fig. 5:**
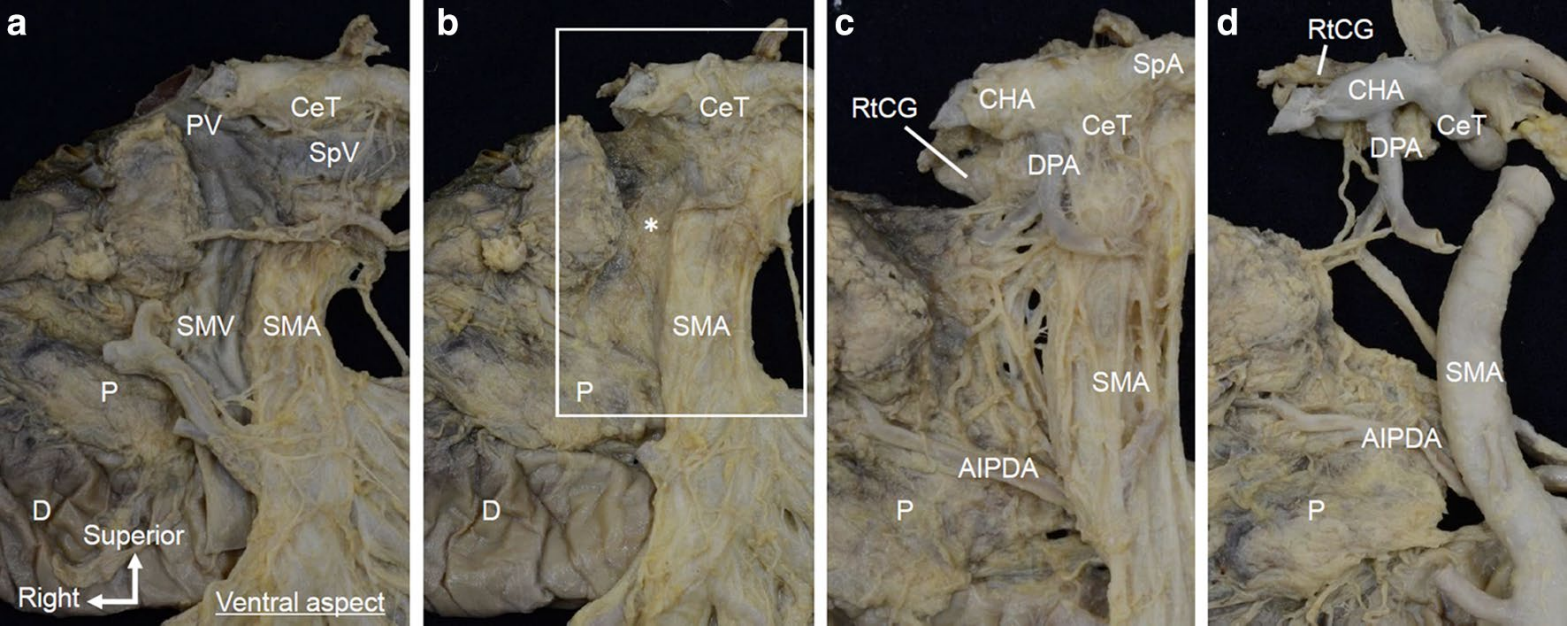
Detailed macroscopic analysis of an isolated en bloc specimen observed from the ventral side. **a** Ventral view of the pancreatic head and the surrounding structures. The PV system, including the PV, SMV, and SpV, was observed. **b** After removal of the PV system, the plate-like structure (*) was observed. It was continuously connected to the uncinate process and CeT/SMA. **c** Magnified view of the white rectangular space of (b) after removing loose connective tissue from the plate-like structure. The fibrous bundles of the plate-like structure included nerves and small vessels and mainly surrounded the CeT and SMA (the celiac plexus and SMA plexus). **d** After removing other arteries (c), arterioles branching from the dorsal pancreatic artery were observed within the plate-like structure. *AIPDA* anterior inferior pancreaticoduodenal artery, *CeT* celiac trunk, *CHA* common hepatic artery, *D* duodenum, *DPA* dorsal pancreatic artery, *P* pancreas, *PV* portal vein, *RtCG* right celiac ganglion, *SMA* superior mesenteric artery, *SMV* superior mesenteric vein, *SpA* splenic artery, *SpV* splenic vein

The isolated en bloc specimens were dissected from the ventral and dorsal sides, and finer structures were observed. The plate-like structure connecting the uncinate process of the pancreas and CeT/SMA was observed on the posterior side of the PV and SMV (Fig. 5a, b). This structure extended continuously from the fibrous tissue around the CeT/SMA to the uncinate process. After removal of loose connective tissue, it was observed that the plate-like structure was composed of abundant fibrous bundles including nerves and small vessels (Fig. 5c). The fibrous bundles mainly surrounded the arteries, such as the CeT and SMA (the celiac plexus and SMA plexus); they were also located between the uncinate process and CeT/SMA. On the right side of the SMA, the uncinate process was attached to the SMA plexus. When the pancreatic head was slightly pulled to the right, the fibrous bundles descended in an arc from the root of the CeT/SMA toward the dorsal side of the distal part of the SMA. That is, fibrous bundles run obliquely between the upper part of the uncinate process and CeT or proximal SMA, between the lower part of the uncinate process and the dorsal side of the distal SMA, which were in an arched curve. Additionally, the plate-like structure included arterioles, such as branches from the dorsal pancreatic artery (Fig. 5d).

**Fig. 6:**
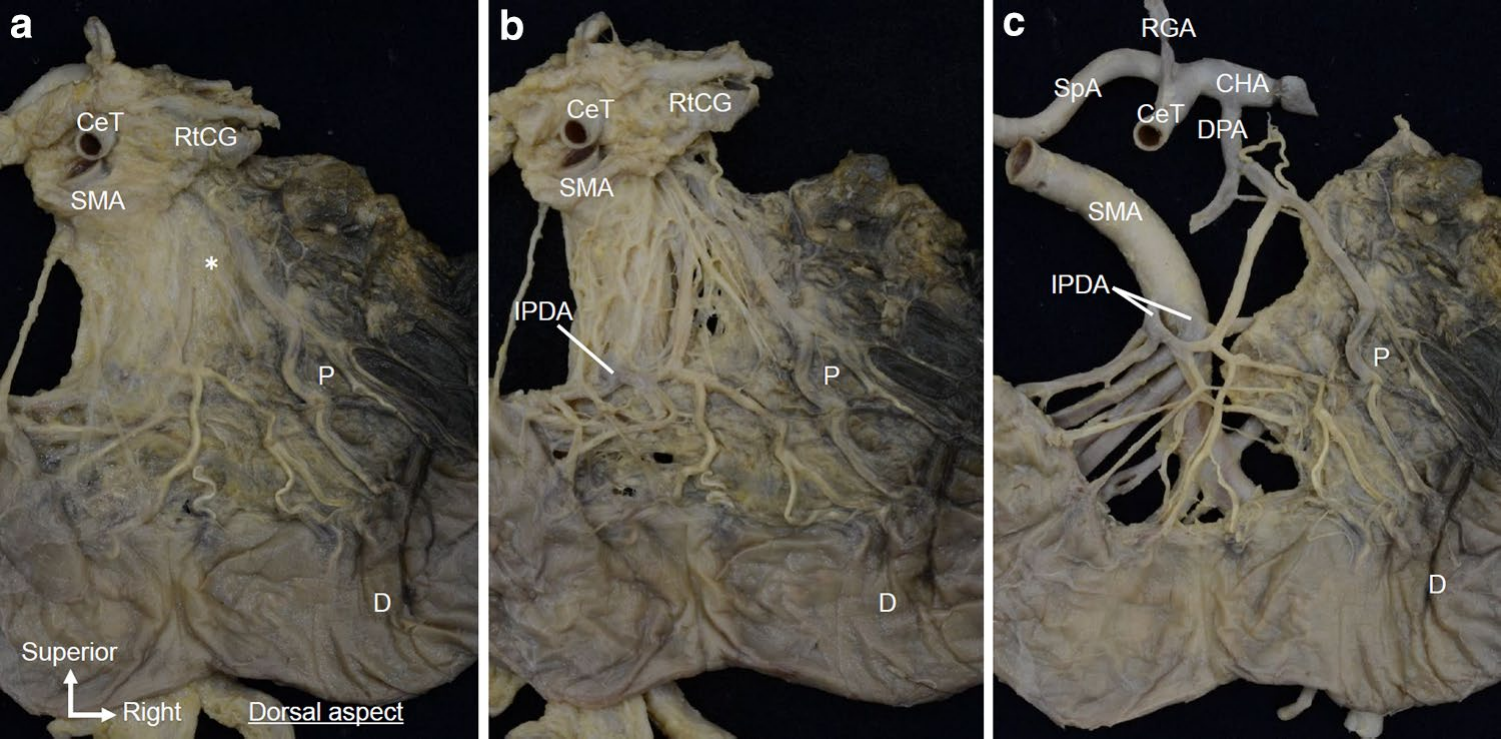
Detailed macroscopic analysis of the isolated en bloc specimen observed from the dorsal side. **a** Dorsal side of the same specimen shown in Fig. 5. The plate-like structure (*) is continuously connected to the RtCG, CeT, SMA, and pancreatic head. **b** After removing the loose connective tissue from the plate-like structure, the fibrous bundles of the plate-like structure were observed to spread radially from the RtCG and the roots of the CeT/SMA toward the pancreatic head. Some fibrous bundles were observed running along the IPDA. **c** After removing other arteries (b), arterioles branching from the dorsal pancreatic artery were observed within the plate-like structure. *CeT* celiac trunk, *CHA* common hepatic artery, *D* duodenum, *DPA* dorsal pancreatic artery, *IPDA* inferior pancreaticoduodenal artery, *P* pancreas, *RtCG* right celiac ganglion, *SMA* superior mesenteric artery, *SpA* splenic artery

When observed from the dorsal side of the same specimen, the plate-like structure spread continuously from the tissue around the right celiac ganglion, CeT, and SMA to the pancreatic head (Fig. 6a). The fibrous bundles of the plate-like structure ran radially from the right celiac ganglion and the roots of the CeT/SMA toward the dorsal side of the pancreatic head and SMA. Furthermore, some fibrous bundles ran along the IPDA and entered the pancreatic head (Fig. 6b). The plate-like structure included arterioles, such as branches from the dorsal pancreatic artery (Fig. 6c).

## Discussion

**Fig. 7:**
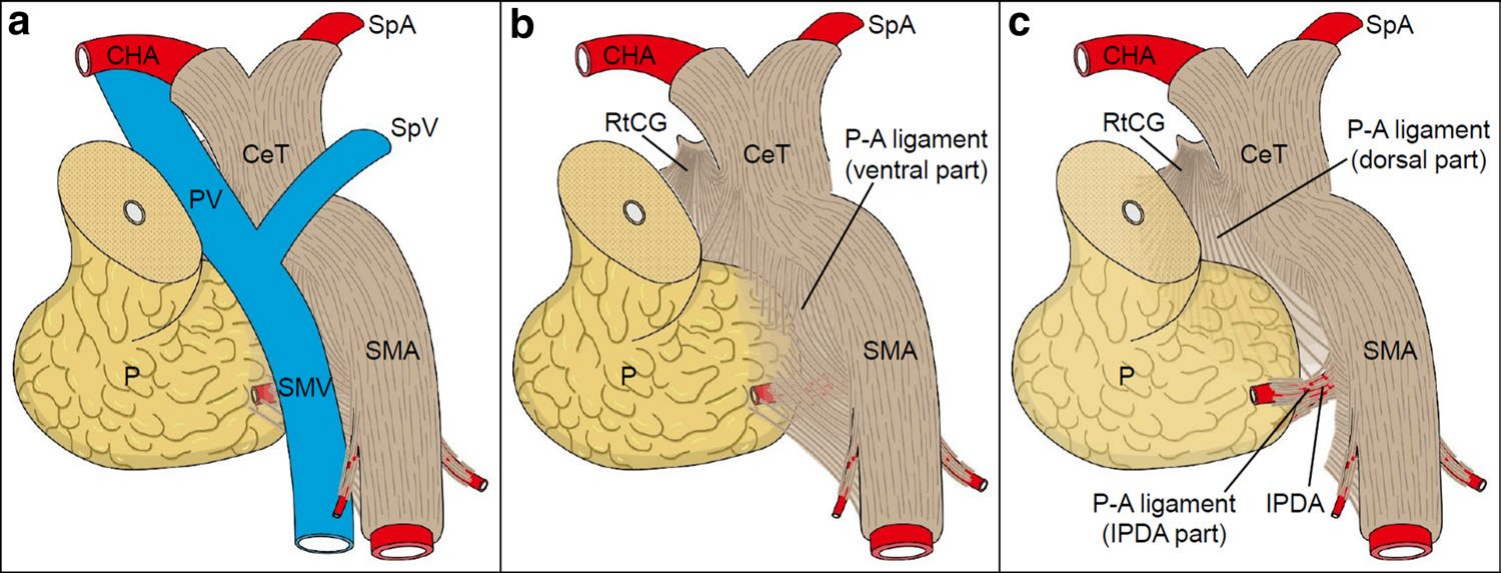
Schematic diagram of the P–A ligament. **a** Ventral view (slightly inferior and right) of the pancreatic head and surrounding structures. The pancreatic head is pulled slightly to the right, as it appears during surgery. **b** Region posterior to the PV/SMV. The P–A ligament is continuously connected to the RtCG, CeT/SMA, and pancreatic head and is composed of macroscopically visible fibrous bundles and loose connective tissue. In the ventral part of the P–A ligament, the uncinate process of the pancreas is attached to the SMA plexus, and the fibrous bundles mainly run along the SMA. **c** View of the region deeper (dorsal) than that in (b). In the dorsal part of the P–A ligament, the fibrous bundles spread radially from the RtCG and CeT toward the pancreatic head and the dorsal side of the SMA. Some fibrous bundles entered the pancreatic head along with the IPDA. *P*–*A* pancreas–major arteries, *CeT* celiac trunk, *CHA* common hepatic artery, *IPDA* inferior pancreaticoduodenal artery, *P* pancreas, *PV* portal vein, *RtCG* right celiac ganglion, *SMA* superior mesenteric artery, *SMV* superior mesenteric artery, *SpA* splenic artery, *SpV* splenic vein

The plate-like structure was observed on the dorsal side of the PV system and was connected to the uncinate process and CeT/SMA (Fig. 7). We propose calling the plate-like structure the P–A ligament because it is positioned between the main arteries branching from the aorta and pancreatic head and it contains fibrous bundles including collagen fibers, adipose tissue, nerve fibers, and small vessels. The P–A ligament consists of three components: the ventral part, the dorsal part, and the IPDA part; each component has different locations and fibrous bundle directions. In the ventral part (superficial layer) of the P–A ligament, the uncinate process is attached to the SMA plexus and the fibrous bundles mainly run along the SMA. In the dorsal part (deep layer) of the P–A ligament, the fibrous bundles spread radially from the right celiac ganglion and CeT toward the dorsal side of the pancreatic head and SMA. Near the IPDA, the fibrous bundles enter the pancreatic head. These three components partly overlap each other.

Previously, the structure between the pancreatic head and the CeT/SMA has been given two main descriptions: “pancreatic head plexus” and “mesopancreas.” Nerves running between the pancreatic head and the CeT/SMA have attracted attention, and the concept of the pancreatic head plexus has been proposed (Yoshioka and Wakabayashi 1985; Yi et al. 2003). From the perspective of perineural invasion, this concept is significant (Nagakawa et al. 1991). However, it was problematic that the concept of the pancreatic head plexus did not describe tissues other than nerves. When the invasion of pancreatic cancer has progressed to a certain stage, it is questionable to only consider the perineural invasion. Furthermore, the term does not clearly include the SMA margin. Therefore, this description would not be useful in the clinical setting. Recently, the concept of the mesopancreas, which includes the tissue between the pancreatic head and the SMA as the mesentery of the pancreas, has been proposed (Gockel et al. 2007; Agrawal et al. 2010; Inoue et al. 2015; Kanhere et al. 2015; Yi et al. 2020). Although this concept is in line with observations during surgery, the original concept by Gockel et al. (2007) was limited to the relationship between the pancreatic head and the SMA. Several reports indicated that the term “mesopancreas” is a misnomer because there is no fascial envelope (Agrawal et al. 2010; Sharma and Isaji 2016). Furthermore, this is a misleading term that implies oncological benefits, despite the lack of an anatomical basis for defining the mesentery. In response to that criticism, Bouassida et al. (2013) defined this area as the “retroportal lamina” as an alternative to the mesopancreas. However, this concept is not based on sufficient anatomical research and is not comprehensive. Moreover, this term is not appropriate because the plate-like structure is not defined by its relationship with the PV system; instead, it is considered to be defined by its relationship with the artery.

As described previously, there is no consensus regarding the anatomy and terminology of the plate-like structure; therefore, we propose that the term “P–A ligament” should be used because it allows for a comprehensive understanding of the anatomy. We observed the plate-like structure in its intact state and analyzed its components. The precise macroscopic examination revealed the running form of the fibrous bundles. To the best of our knowledge, this is the first study to describe the form of the fibrous bundles that run obliquely between the lower part of the uncinate process and the dorsal side of the distal SMA. Furthermore, the histological examination clarified that the fibrous bundles included collagen fibers, adipose tissue, nerves, and vessels. The term “ligament” is derived from the Latin “ligamentum,” which means a connecting or unifying bond. Therefore, the term “P–A ligament” is a more comprehensive description than those previously used (Table 1).

**Table 1:** Definitions of the plate-like structure located between the pancreatic head and the celiac trunk/superior mesenteric artery

Year	Authors	Terms		Components	
				Macroscopic	Histologic
1958	Yoshioka and Wakabayashi	PLph1	PLph2	Nerves	–
2003	Yi et al.	Posterior hepatic plexus	Nerve along the IPDA	Nerves	–
2007	Gockel et al.	–	Mesopancreas	–	Nerves, lymphatic vessels
2013	Bouassiada et al.	–	Retroportal lamina	–	Areolar tissue, adipose tissue, nerves, vessels
Present study		P–A ligament		Fibrous components, loose connective tissue	Collagen fibers, adipose tissue, nerves, vessels

*IPDA* inferior pancreaticoduodenal artery, *P*–*A* ligament pancreas–major arteries ligament

The running form of the fibrous bundles of the P–A ligament differed depending on their location. This difference in fiber directions can be understood by the positional relationship between the pancreatic head and the arteries. The running pattern of fibrous bundles in the ventral part of the P–A ligament seems to be strongly influenced by the SMA. The fibrous bundles do not run transversely and do not flow vertically into the uncinated process; instead, they mainly flow superiorly to inferiorly along the SMA. Therefore, it may be appropriate to consider that the uncinate process is incorporated in the flow of the SMA plexus. However, the radial running pattern of the fibrous bundles in the deep part of the P–A ligament seems to reflect the origin point localized near the right celiac ganglion and the roots of the CeT and SMA. The fibrous bundles exhibit a radial running pattern, probably because the fibers originating from the dorsal location spread widely throughout the dorsal side of the pancreatic head. Furthermore, fibrous bundles run along the IPDA. This running pattern suggests that the fiber arrangement is strongly affected by the arteries.

The P–A ligament is the plate-like structure continuously connected to the pancreatic head and CeT/SMA that contains fibrous bundles including collagen fibers, adipose tissue, nerve fibers, and small vessels. It comprises three components—the ventral part, dorsal part, and IPDA part—which have characteristic running patterns depending on their location. The “P–A ligament” may be a clinically useful term that allows for a comprehensive understanding of the anatomy around the pancreatic head and that is expected to provide an anatomical basis for further discussions of pancreatic surgery.

## Data Availability

All relevant data used this study can be accessed upon reasonable request to the corresponding author.
